# Inflammatory and cytotoxic responses of an alveolar-capillary coculture model to silica nanoparticles: Comparison with conventional monocultures

**DOI:** 10.1186/1743-8977-8-6

**Published:** 2011-01-27

**Authors:** Jennifer Kasper, Maria I Hermanns, Christoph Bantz, Michael Maskos, Roland Stauber, Christine Pohl, Ronald E Unger, James C Kirkpatrick

**Affiliations:** 1 University Medical Centre, Institute of Pathology, Mainz, Germany; 2 Johannes Gutenberg University Mainz, Institute of Physical Chemistry, Mainz, Germany; 3 BAM Bundesanstalt für Materialforschung und -prüfung, Berlin, Germany; 4 University Medical Centre, Molecular and Cellular Oncology, ENT Department, Mainz, Germany

## Abstract

**Background:**

To date silica nanoparticles (SNPs) play an important role in modern technology and nanomedicine. SNPs are present in various materials (tyres, electrical and thermal insulation material, photovoltaic facilities). They are also used in products that are directly exposed to humans such as cosmetics or toothpaste. For that reason it is of great concern to evaluate the possible hazards of these engineered particles for human health. Attention should primarily be focussed on SNP effects on biological barriers. Accidentally released SNP could, for example, encounter the alveolar-capillary barrier by inhalation. In this study we examined the inflammatory and cytotoxic responses of monodisperse amorphous silica nanoparticles (aSNPs) of 30 nm in size on an *in vitro *coculture model mimicking the alveolar-capillary barrier and compared these to conventional monocultures.

**Methods:**

Thus, the epithelial cell line, H441, and the endothelial cell line, ISO-HAS-1, were used in monoculture and in coculture on opposite sides of a filter membrane. Cytotoxicity was evaluated by the MTS assay, detection of membrane integrity (LDH release), and TER (Transepithelial Electrical Resistance) measurement. Additionally, parameters of inflammation (sICAM-1, IL-6 and IL-8 release) and apoptosis markers were investigated.

**Results:**

Regarding toxic effects (viability, membrane integrity, TER) the coculture model was less sensitive to apical aSNP exposure than the conventional monocultures of the appropriate cells. On the other hand, the *in vitro *coculture model responded with the release of inflammatory markers in a much more sensitive fashion than the conventional monoculture. At concentrations that were 10-100fold less than the toxic concentrations the apically exposed coculture showed a release of IL-6 and IL-8 to the basolateral side. This may mimic the early inflammatory events that take place in the pulmonary alveoli after aSNP inhalation. Furthermore, a number of apoptosis markers belonging to the intrinsic pathway were upregulated in the coculture following aSNP treatment. Analysis of the individual markers indicated that the cells suffered from DNA damage, hypoxia and ER-stress.

**Conclusion:**

We present evidence that our *in vitro *coculture model of the alveolar-capillary barrier is clearly advantageous compared to conventional monocultures in evaluating the extent of damage caused by hazardous material encountering the principle biological barrier in the lower respiratory tract.

## Background

Over the past 10 years nanoparticulate material has gained tremendously in importance for industrial applications. Synthetic non-metal amorphous silica (SiO_2_) nanoparticles (aSNPs) are processed in a variety of industrial products, e.g. photovoltaic, tyre compounds or electrical and thermal insulation material. They are also a component of products which are directly exposed to humans, e.g. cosmetics or toothpaste [1,2]. Hence humans are variously exposed to silica nanomaterials. Inhalation of higher doses of silica nanomaterials can particularly occur in occupations such as ceramic, mine or foundry workers, dental laboratory technicians, agricultural workers or stone cutters.

Silica appears in two different forms: 1. The crystalline form which is arranged in tetrahedral SiO_4 _units (such as α-quartz, α-tridymite etc.) 2. The amorphous form which does not have such a long-range order of the positions of the atoms. Since the health hazards of the crystalline form, which among other things causes silicosis, have been well documented [3] for the micrometre scale, the amorphous form has been considered as having low toxicity. Therefore, aSNPs are an FDA-approved food additive [4]. Furthermore, aSNPs are used as a low toxic reference material in cytotoxicity experiments [5]. However, several cytotoxicity studies on nanoscaled aSNPs indicate a possible serious hazard for human health. Napierska et al. [6] and Lison et al. [7] demonstrated via MTT and LDH assays a concentration-dependent cytotoxicity of aSNP on an endothelial cell line EA.hy926, an epithelial cell line A549 and on monocyte-macrophages J774. Additionally, Waters et al. [1] employed a macrophage cell model to demonstrate that non-opsonised aSNPs stimulate inflammatory protein secretion such as RANTES, TNF-α, VEGF and G-CSF.

Nanoparticles (NPs) could enter the body by passing through biological diffusion barriers such as the skin (epidermis), the gastrointestinal tract (gastrodermis) or the lung (respiratory epithelium) i.e. the alveolar-capillary barrier. Physiologically, biological barriers are designed to handle the interplay of two different aspects. On the one hand they allow the passage of external substances that are essential for the organism (e.g. gas exchange at the alveolar-capillary barrier), while on the other hand biological barriers protect the body from hazardous exterior substances. Hence, research addressing the toxicity of industrially fabricated NPs or the suitability of particles specially synthesized for drug and gene delivery should focus on the interaction of these NPs with biological barriers.

Our studies were focussed on the toxicity of aSNPs exposed to the lower respiratory tract i.e. the alveolar-capillary unit. The alveolar region of the lung has a very large surface area (100-140 m^2^) [8], which makes it an interesting target for drug and gene delivery, but at the same time represents a serious point of attack for harmful nanomaterials. In *in vivo *studies this alveolar-capillary barrier is difficult to access. We therefore developed a coculture model that mimics the alveolar-capillary barrier *in vivo *[9]. The model consists of a human lung epithelial cell line with characteristics of type II pneumocytes and Clara cells (NCI H441) [10] and the human microvascular endothelial cell (MEC) line, ISO-HAS-1 [11,12], on opposite sides of a transwell filter unit. With a cultivation time of 10 days in coculture both cells differentiate to polarized, barrier-forming cells. They develop circumferential cell-cell contacts expressing the tight junctional (TJ) protein, ZO-1, and the adherens junction (AJ) proteins, E-Cadherin, as well as α- and ß-Catenin. Additionally, they show trans-bilayer electrical resistance (TER) values that average 560 ± 6 Ω * cm^2^. In the present study we compared the properties of two different cell culture systems after aSNP exposure: 1. the conventional monoculture, in which the cells are seeded on tissue culture plastic 24 or 48 h prior to the experiment 2. the monoculture on transwell filter supports that allows the cells to reach a more differentiated state by establishing functional barrier properties and 3. coculture models of the alveolar-capillary barrier that allow the cross-talk between the two main barrier forming cells, the epithelium and the endothelium. Our study demonstrates that not only the polarized nature of cells on transwell filter supports but also the coculture with adjacent cells in close proximity play an important role in the effects caused by aSNP exposure. Compared to conventional monocultures we show that the latter model more closely mimics the *in vivo *situation and therefore should have a greater predictive function concerning effects of nanoparticulate matter contacting the lower respiratory tract.

## Methods

### Characterization of amorphous silica nanoparticles (aSNPs)

In this study two aSNPs with similar sizes from different companies were used to corroborate a material-specific but not a fabrication-specific response of the cells on aSNP treatment. The Ludox TM-40 silica particles were purchased from Sigma-Aldrich (St. Louis, MO, USA) and the NexSil20 aqueous silica dispersion was obtained from Nyacol Nano Technologies, Inc. (Ashland, MA, USA). All nanoparticles were characterized with respect to shape, size and size distribution in the dry state as well as in solution. Transmission Electron Microscopy (TEM) imaging was performed using a Philips EM420, operating at 120 kV, on carbon-coated copper grids. The apparent hydrodynamic diameter (*D*_*h*_)_*z *_was determined by Dynamic Light Scattering (DLS), both in a ready-to-use instrument (Malvern Zetasizer Nano ZS, single angle measurements at 173°, He-Ne-Laser with λ = 633 nm) and a fully equipped setup with a Coherent Verdi V2 diode-pumped solid-state laser (λ = 532 nm), an ALV-SP125 goniometer with single photon detector SO-SIPD and an ALV-5000 Multiple-Tau digital correlator. Angle-dependent measurements were carried out between 30° and 150° and the data were evaluated by exponential fitting and q = 0 extrapolation; μ_2 _values as an estimate for the polydispersity of the sample were determined at 90° (assuming a Gaussian distribution, a μ_2 _value of <0,04 approximately corresponds to a standard deviation of <28%) [13].

Additionally to the DLS measurements, the zeta potential of the particles was measured with the Zetasizer (measuring angle for zeta potential: 17°) to study the aggregation behaviour of the nanoparticle dispersions.

### Cell culture

#### Monocultures of ISO-HAS-1 and H441

Prior to seeding cells the 96-well plates (TPP Switzerland) were coated with 50 μl fibronectin for 1 h at 37°C (5 μg/ml, Roche Diagnostics, Mannheim). The human microvascular endothelial cell line, ISO-HAS-1 [11,12], and the human lung adenocarcinoma cell line, H441 (purchased from ATCC, ATCC-HTB-174, Promochem, Wesel, Germany), were used in these experiments. The cells were seeded (ISO-HAS-1: 1.6 × 10^4 ^cells/well, H441: 3.2 × 10^4 ^cells/well) from a confluent culture flask on 96-well plates in RPMI 1640 medium (Gibco) with L-glutamine supplemented with 10% FCS and Pen/Strep (100 U/100 μg/ml) and cultivated at 37°C, 5% CO_2 _for 24 h prior to SNP exposure to a confluent layer of cells.

#### Coculture of ISO-HAS-1 with H441

The procedure of coculture was performed as described previously [14] with some alterations. Briefly, HTS 24-Transwell^® ^filters (polycarbonate, 0.4 μm pore size; Costar, Wiesbaden, Germany) were coated with rat tail collagen type-I (12.12 μg/cm^2^, BD Biosciences, Heidelberg, Germany). ISO-HAS-1 cells (1.6 × 10^4^/well ≙ 5 × 10^4^/cm^2^) were seeded on the lower surface of the inverted filter membrane. After 2 h of adhesion at 37°C and 5% CO_2_, H441 (8.4 × 10^3^/well ≙ 2 × 10^4^/cm^2^) were placed on the top side of the membrane. The cells were cultured for about 10 days in RPMI 1640 medium with L-glutamine supplemented with 5% FCS, Pen/Strep (100 U/100 μg/ml). From day 3 of cultivation the H441 were treated with dexamethasone (Dex: 1 μM). When stimulated with dexamethasone, the cocultures of NCI H441 and ISO-HAS-1 establish contact-inhibited differentiated monolayers. Concerning barrier properties, a maximum of 565 ± 48 Ω*cm^2 ^can be attained for the coculture under treatment with Dex on day 11 of cocultivation. The NCI H441 show a continuous, circumferential immunostaining of the tight junctional protein, ZO-1 and the adherens junction protein, E-cadherin. However, experiments regarding cytokine release may be compromised to a certain extent under Dex treatment. Recent internal studies revealed a decreased elevation of the growth factor VEGF upon TNF-α stimulation of dexamethasone-treated H441 compared to untreated cells (data not shown). Thus, the dexamethasone concentration was finely adjusted to combine functional barrier properties on the one hand and a functional cytokine network on the other hand. As a control, monocultures of H441 or ISO-HAS-1 were seeded on transwells and kept under the same culture conditions as described for the cocultures. Primary cocultures were prepared as described previously by Hermanns et al. [15].

### Exposure to aSNP

To avoid nanoparticle aggregation predilutions of the aSNP dispersion were made in pure water (Braun ad iniectabilia, Braun Melsungen AG, Melsungen). Due to nanoparticle aggregation in serum-containing medium, serum-free medium was used during 4 h exposure. All dilutions were applied 1:10 in serum-free RPMI to the cells (10 μl NP-dispersion + 90 μl RPMI). Since both culture systems (mono- and coculture) have the same culture area/well, the volume of the applied aSNP dispersion was adjusted to 100 μl in both systems. After an exposure time of 4 h the cells were either evaluated or washed twice with RPMI and cultured for a further 20 h period. After 4 h and 24 h recovery cell cytotoxicity was studied by various assays. For the coculture aSNPs were exclusively applied to the apical side of the H441 layer on top of the 24 well transwells. To study exposure conditions as they might occur for humans, either by work place or exposure after an accidental event, a relatively wide concentration range of 0.6 μg to 6000 μg/ml aSNP was chosen. A concentration of 6000 μg/ml might not mimic realistic inhalative exposure conditions as they might occur, for example, in the case of a ceramic worker. Nevertheless, this high concentration of aSNPs was included as a positive control with a significant cytotoxic effect observed in our mono- and cocultures.

### Endotoxin remnants in aSNP dispersions

Prior to all experiments the particles were checked for endotoxin contamination by E-selectin induction on endothelial cells via ELISA (Duoset ELISA Kit (R&D Systems^®^, Wiesbaden, Germany) and immunofluorescence. For the ELISA-assay cells were seeded on 96-well plates and exposed to aSNPs as described above. The supernatant was taken after 24 h. As positive control cells were incubated with TNF-a (300 U/ml ≅ 0.732 g/ml) or lipopolysaccharide from E.coli (LPS, 1 μg/ml, Sigma). For the immunocytochemical E-selectin staining cells were seeded (ISO-HAS-1: 8 × 10^4 ^cells/well) on Lab-Tek™ chamberslides (Nunc, Wiesbaden, Germany) and exposed to aSNPs for 4 h as described above. Subsequently, they were fixed with paraformaldehyde (3.7%) in CS buffer (PIPES 0.1 M, EGTA 1 mM, 4% polyethylene glycol 800, NaOH 0.1 M) for 20 min at room temperature, and washed three times. Cell membranes were then permeabilized with 0.2% Triton X-100 in PBS for 10 minutes. After washing three times in PBS cells were stained with primary antibodies (in PBS + 1% BSA for 1 h at room temperature) obtained from Monosan (CellSystems, St. Katharinen, Germany). The secondary antibody (Alexa fluor 488-conjugated, anti-mouse) was added for 1 h after washing three times in PBS. Terminal cells were counterstained with Hoechst 33342 (Sigma) and mounted with Fluoromount-G™ (SouthernBiotech). Visual examination was conducted by means of a fluorescent microscope (personalDV, Applied Precision, Issaquah, USA).

### Studies on cytotoxicity

On the cell layer CellTiter 96^® ^AQ_ueous _One Solution Cell Proliferation Assay (MTS) was used to study cytotoxicity of aSNPs. At aSNP concentrations of 600 μg/ml or higher, interference with the MTS reagent occured. To avoid false positive results due to particle-dye interactions supernatant (in total 100 μl) was collected after aSNP exposure and saved at -20°C. Thereafter, the cells were washed with PBS and incubated with fresh medium containing MTS reagent (dilution 1:10) for 1 h before absorbance measurements at 490 nm.

50 μl of the collected supernatant was used in the CytoTox 96^® ^Non-Radioactive Cytotoxicity Assay (Promega, Mannheim, Germany) to determine lactate dehydrogenase (LDH) release due to membrane disruption. This enzyme is present in the cytosol and catalyses the lactate-to-pyruvate reaction. Disruption of plasma membrane integrity leads to a release of LDH into the supernatant, resulting in the conversion of a tetrazolium salt into a red formazan product (absorbance at 490 nm).

In the LDH assay for the coculture (4 h exposure) concentrations of 6000 μg/ml aSNP showed false positive results as a result of particle-dye interactions (Ludox TM-40: 10 ± 1.6%, NexSil20: 10 ± 0.66% of the lysis control). Therefore, the percentage due to aSNP interference has been subtracted from the measurements.

### Release of inflammatory mediators

50 μl of the supernatant taken after 4 h SNP exposure was analysed for soluble intercellular adhesion molecule-1 (sICAM-1) and inflammatory cytokine release (IL-6, IL-8) using Duoset ELISA Kits (R&D Systems^®^, Wiesbaden, Germany). Additionally, after removal of the unbound aSNPs cells were cultured for further 20 h in RPMI with 10% fetal calf serum and further tested for sICAM-1 and cytokine release.

### Transbilayer Electrical Resistance Measurements

To evaluate the functional efficiency of an intact barrier *in vitro *the transepithelial electrical resistance (TER) was measured with an EVOM voltohmmeter (World Precision Instruments, Berlin, Germany) equipped with a STX-2 chopstick electrode. HTS 24-Transwell^® ^filter membranes without cells coated with rat tail collagen type-I were measured and set as blank (approximately 110 Ω). Barrier resistance readings (Ω) were obtained for each well individually and, after subtracting the resistance of the blank filter membrane, were multiplied by the membrane area (0.33 cm^2^) to give Ωcm^2^. In the experiments showing a time-dependent effect of aSNP exposure the TER is expressed as % of t_0 _(TER value before aSNP exposure).

### Immunofluorescence

Immunofluorescence was performed to detect morphological changes following aSNP exposure. Subsequent to aSNP treatment cells were fixed with paraformaldehyde (3.7%) in CS buffer (PIPES 0.1 M, EGTA 1 mM, 4% polyethylene glycol 800, NaOH 0.1 M) for 20 min at room temperature, and washed three times. Cell membranes were then permeabilized with 0.5% Triton X-100 in PBS for 10 minutes. After washing three times in PBS epithelial cells were stained with primary antibodies such as E-Cadherin (in PBS + 1% BSA for 1 h at room temperature) obtained from Monosan (CellSystems, St. Katharinen, Germany), endothelial cells were stained for anti-human PECAM-1 from Dako (Hamburg, Germany). The secondary antibody (Alexa fluor 488 or 546-conjugated, anti-mouse) was added for 1 h after washing three times in PBS. Cell nuclei were stained with Hoechst 33342 (Sigma) and samples were mounted with Fluoromount-G™ (SouthernBiotech). Pictures were taken by means of a fluorescent microscope (personalDV, Applied Precision, Issaquah, USA).

### Apoptosis marker analysis

To detect the production of 35 apoptosis-related proteins upon aSNP exposure the Human Apoptosis Array Kit (Proteome Profiler™ Array, ARY009 from R&D Systems^®^, Wiesbaden, Germany) was used. After 10 min, 1 hour and 4 hour exposure to 600 μg/ml NexSil20 the transwell filters were cut, transferred into the lysis buffer for 30 min at 4°C and mixed at 60 rpm using the Intelli-Mixer (NeoLab, Heidelberg, Germany). Four filters with the same exposure conditions were mixed and for the array two similar experiments were pooled. Thus, eight identically treated cocultures were analyzed. After lysis, protein content was quantified using the BCA-Kit (Pierce, Schwerte, Germany). The absorption at 550 nm was measured in a plate reader (Titertek Multiskan Plus; Labsystems, Frankfurt, Germany). In the Human Apoptosis Array Kit 200 μg protein was used per sample. Further procedures were performed according to the manufacturer's protocol. The resulting spots were visualized via chemoluminescence detected by means of the Chemi-Smart 5100 (peqlab, Erlangen, Germany). A detailed evaluation of chemoluminescence intensity was conducted using the software Array-Pro Analyzer Version 4.5 from Media Cybernetics (Bethesda, USA).

### Statistical analysis

From several independent measurements, means and standard deviations were calculated. Analyses are shown as mean ± S.D. from at least three separate experiments. Testing for significant differences between means was carried out using one-way ANOVA and Dunnett`s Multiple Comparison test at a probability of error of 5% (*), 1% (**) and 0.1% (***).

## Results

### Characterization of amorphous silica nanoparticles (aSNPs)

Prior to the experiments we examined the stability of the nanoparticle dispersions in Milli-Q water, phosphate buffered saline (PBS, 150 mM salt in total) and the cell culture medium RPMI 1640 (Table 1).

**Table 1:** The shown measurements were performed 20 minutes after preparation of the samples at concentrations of 600 μg/ml (corresponding particle concentration: 4.4·10^13 ^particles per ml dispersion) and a temperature of 20°C.

Diluent	aSNP (10% + 90% diluent)	
	**NexSil20**	**Ludox TM-40**
**(*D***_***TEM***_**)/nm**	**31.4 ± 3.8**	**31.2 ± 4.0**

**Milli-Q water**:		
**ζ/mV**	-51.0	-57.3
**(*D***_***h***_**)**_***z***_**/nm**	32.8 | 35.1	32.0 | 37.9
***μ***_***2***_	0.12 | 0.04	0.06 | 0.03
**PBS**:		
**ζ/mV**	-18.2	-20.0
**(*D***_***h***_**)**_***z***_**/nm**	36.8 | 37.2	34.2 | 32.6
***μ***_***2***_	0.08 |0.17	0.04 | 0.01
**Medium RPMI1640**:		
**ζ/mV**	-19.8	-26.6
**(*D***_***h***_**)**_***z***_**/nm**	35.4 | 33.4	32.2 | 32.6
***μ***_***2***_	0.07 | 0.03	0.02 | 0.01

The first values of the size measurements were determined with the fully equipped DLS setup, the second ones with the Zetasizer; the corresponding μ_2 _values diverge because of the different measuring angles

The size of the nanoparticles in the dry state (*D*_*TEM*_) was nearly the same as in solution, indicating that the effect of particle shrinking during the preparation of the samples for TEM is quite small (see additional file 1, Figure S1). In all media the hydrodynamic diameter of the particles remained at a value of about 33 nm (for both particle types). The formation of aggregates would be clearly identified by the angular-dependent correlation function as determined by DLS, but was not detected in any case here. Additionally, already a minor fraction of aggregated particles would increase the scattering intensity dramatically (scaling with *D*^*6*^), which would directly lead to an increased radius - which was not observed either. Moreover, no marked change in particle size was determined on measuring 20 min, 140 min, 260 min and 24 h after preparation; even at different temperatures (20° and 37°C) only minor differences were detected. Applying the Ludox TM-40 sample to the cell culture medium, it showed a doubling of the polydispersity after 24 hours at nearly constant size, pointing to a slightly increased tendency of the particles to aggregate. Within the given limitations of a ready-to-use instrument like the Zetasizer and for the investigated system, the results using the different DLS equipment were identical. Thus, the values of the Zetasizer (second column) could be confirmed by the more precise measurements.

With increasing concentration of salt and organic additives, the zeta potential of the particles became less negative - in contrast to the fact that the size remained stable. Even if the ionic strength was not yet high enough to affect the stabilisation through surface charges, this additionally indicates at least an increased tendency to form aggregates.

#### Endotoxin remnants in aSNP dispersions

Prior to all experiments the particles were checked for endotoxin contamination via ELISA and immunofluorescence for E-selectin induction on endothelial cells. None of the used particles induced an E-selectin signal in endothelial cells after 4 and 24 h exposure (Data not shown).

The induction of E-Selectin in endothelial cells is a highly sensitive method similar to the classical Limulus amebocyte lysate test with a sensitivity of less than 3 pg/ml LPS. This means that there is no detectable endotoxin contamination in both aSNPs samples which might interfere with all subsequent tests.

#### Viability after aSNP exposure on monocultures (MTS)

**Figure 1:**
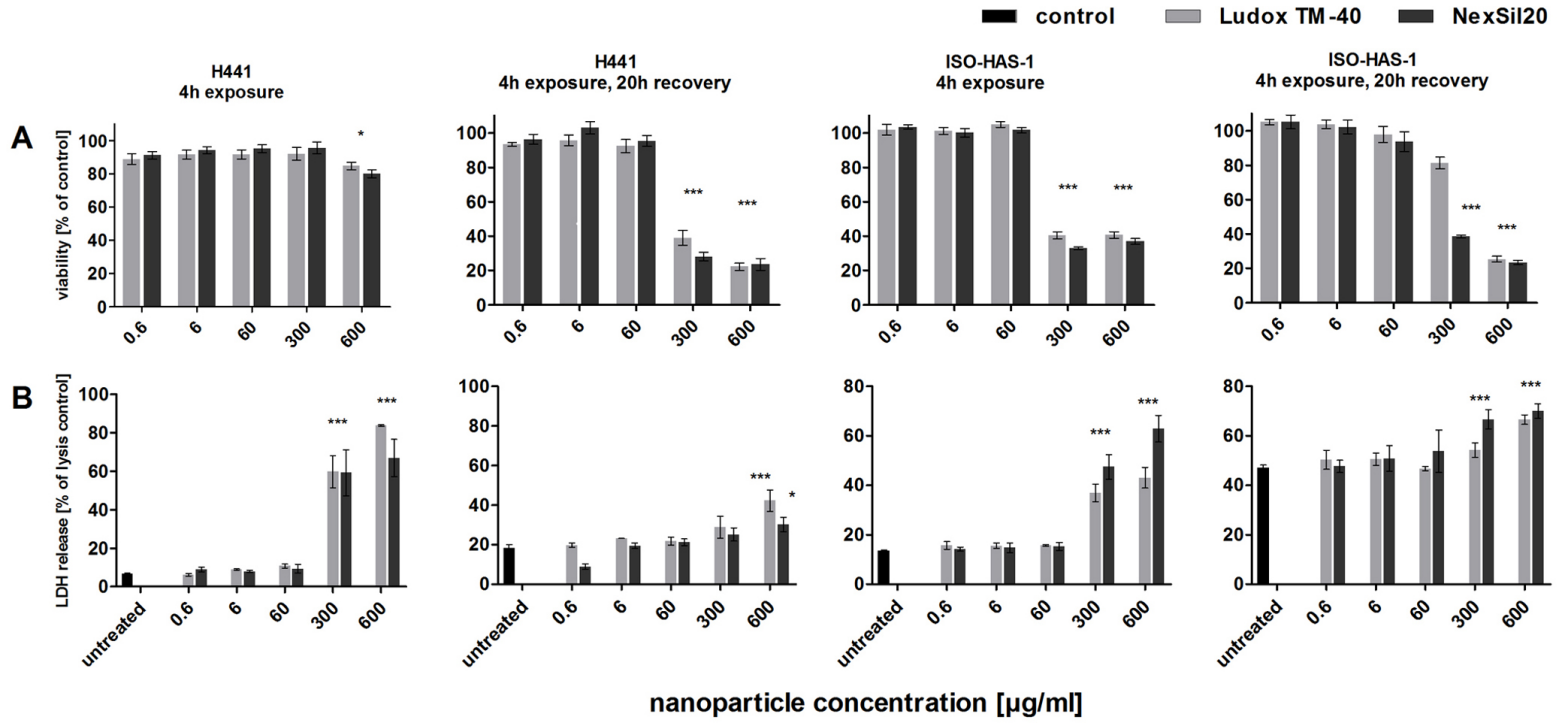
**Mitochondrial activity was measured via the MTS assay (A) and membrane integrity was determined by the LDH assay (B) for monocultures of H441 and ISO-HAS-1 on 96 well plates (conventional monoculture)**. Cells were incubated with aSNP (Ludox TM-40: light grey, NexSil20: dark grey) for 4 h in serum-free medium. aSNPs were then removed and cells were cultivated for further 20 h. The assays were conducted after both time points (4 h exposure and 4 h exposure with 20 h recovery). Data are depicted as percentage of the untreated control (A: MTS) or as percentage of the total LDH amount of the cells (B: LDH, lysis control). Results are shown as means ± S.D. (n = 6-9) of 2-3 independent experiments. *P < 0.05, **P < 0.01 and ***P < 0.001 compared to the untreated control

Both particles NexSil20 and Ludox TM-40 at a concentration of 600 μg/ml (180 μg/cm^2 ^cell layer) resulted in a cell viability of about 80% of the untreated control cells (Ludox TM-40: 85 ± 8.4% and NexSil20: 80 ± 7.9%) for the H441 cell line and about 40% (Ludox TM-40: 41 ± 6.5% and NexSil20: 37 ± 6%) for ISO-HAS-1 (MTS assay, Figure 1A) in the conventional monoculture after 4 h incubation. 300 μg/ml aSNP showed no significant effect on the viability of H441 (Ludox TM-40: 97 ± 8.4% and NexSil20: 95 ± 7%), whereas for the ISO-HAS-1 cells a reduced viability (Ludox TM-40: 32 ± 1.3% and NexSil20: 40 ± 3.5%) was still measurable. Thus, the endothelial cell line seems to be more sensitive to aSNP exposure than the epithelial cell line. After removal of the unattached aSNPs cell viability further decreased in a concentration-dependent fashion for both cell lines (see Figure 1A: 4 h exposure, 20 h recovery). A concentration of 60 μg/ml aSNPs or less did not show any effect on MTS conversion.

#### Membrane integrity (LDH release) upon aSNP stimulation

The LDH assay provided corresponding results to the viability test of the monocultures (Figure 1B). H441 and ISO-HAS-1 cells showed a concentration-dependent release of LDH. After 4 h exposure to 600 μg/ml aSNPs H441 cells released about 75% (Ludox TM-40: 78 ± 14% and NexSil20: 74 ± 17%) of their total LDH, whereas ISO-HAS-1 cells released less LDH (Ludox TM-40: 44 ± 7% and NexSil20: 62 ± 5%) into the supernatant. 300 μg/ml aSNPs also induced a significant loss of LDH in H441 (Ludox TM-40: 53 ± 11% and NexSil20: 59 ± 11%) and ISO-HAS-1 (Ludox TM-40: 37 ± 9% and NexSil20: 47 ± 4.5%). The lower LDH content of the supernatant measured for 300 and 600 μg/ml aSNP in H441 after 20 h recovery compared to 4 h (Figure 1B) is due to the fact that after 4 h medium was replaced. Hence, after medium change a minor membrane leakage was observed for H441, whereas ISO-HAS-1 continuously released LDH to the culture supernatant during the 20 h recovery in a concentration-dependent manner.

**Figure 2:**
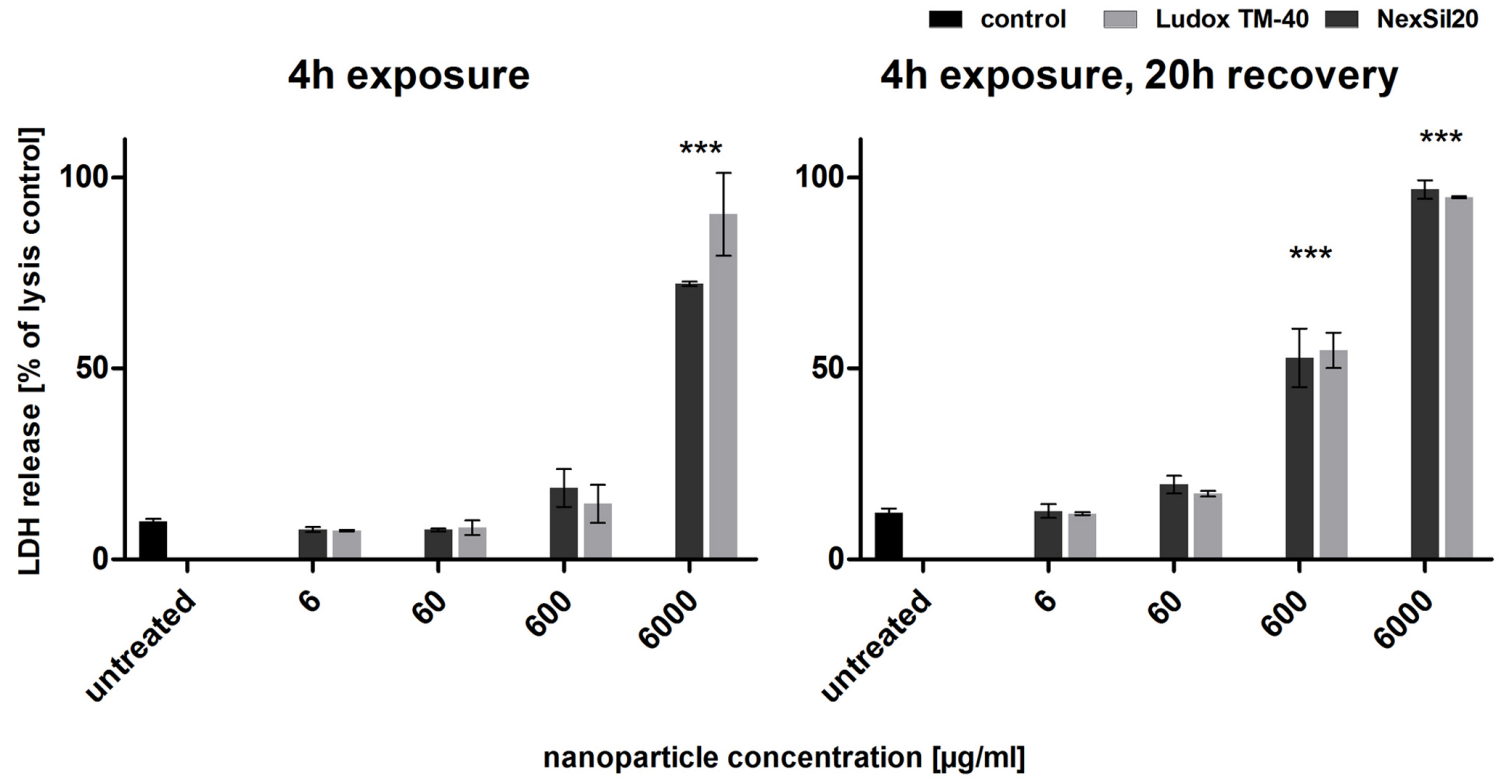
**Membrane integrity was determined by the LDH assay for H441 in coculture**. The H441 of the coculture were incubated with aSNP (Ludox TM-40: light grey, NexSil20: dark grey) for 4 h in serum-free medium. aSNPs were then removed and cells were cultivated for further 20 h. The assays were conducted after both time points (4 h exposure and 4 h exposure with 20 h recovery). Data are depicted as percentage of the total LDH amount of the cells (lysis control). Results are shown as means ± S.D. (n = 6-9) of 2-3 independent experiments. *P < 0.05, **P < 0.01 and ***P < 0.001 compared to the untreated control

An LDH leakage was also measured after 4 h apical (upper well) exposure and also 20 h recovery of the coculture (Figure 2). After 4 h a concentration of 600 μg/ml aSNPs caused a non-significant release of LDH (Ludox TM-40: 19 ± 10% and NexSil20: 15 ± 5%), whereas after 20 h recovery a significant, but much lower release compared to the monoculture, was detected for 600 μg/ml aSNPs, apically exposed (Ludox TM-40: 53 ± 11% and NexSil20: 35 ± 5%). Incubation with 6000 μg/ml, however, showed a considerable LDH release at 4 h (Ludox TM-40: 72 ± 0.85% and NexSil20: 90 ± 11%) as well as after 20 h recovery (Ludox TM-40: 97 ± 3.5% and NexSil20: 95 ± 0.24%). The coculture revealed a lower responsiveness towards aSNP exposure concerning LDH release. Compared to the conventional monocultures the response towards aSNP treatment was shifted by about a factor of 10 of aSNP concentration (from 600 μg/ml to 6000 μg/ml).

#### Comparison of inflammatory responses of monocultures (conventional and differentiated) and differentiated cocultures

**Figure 3:**
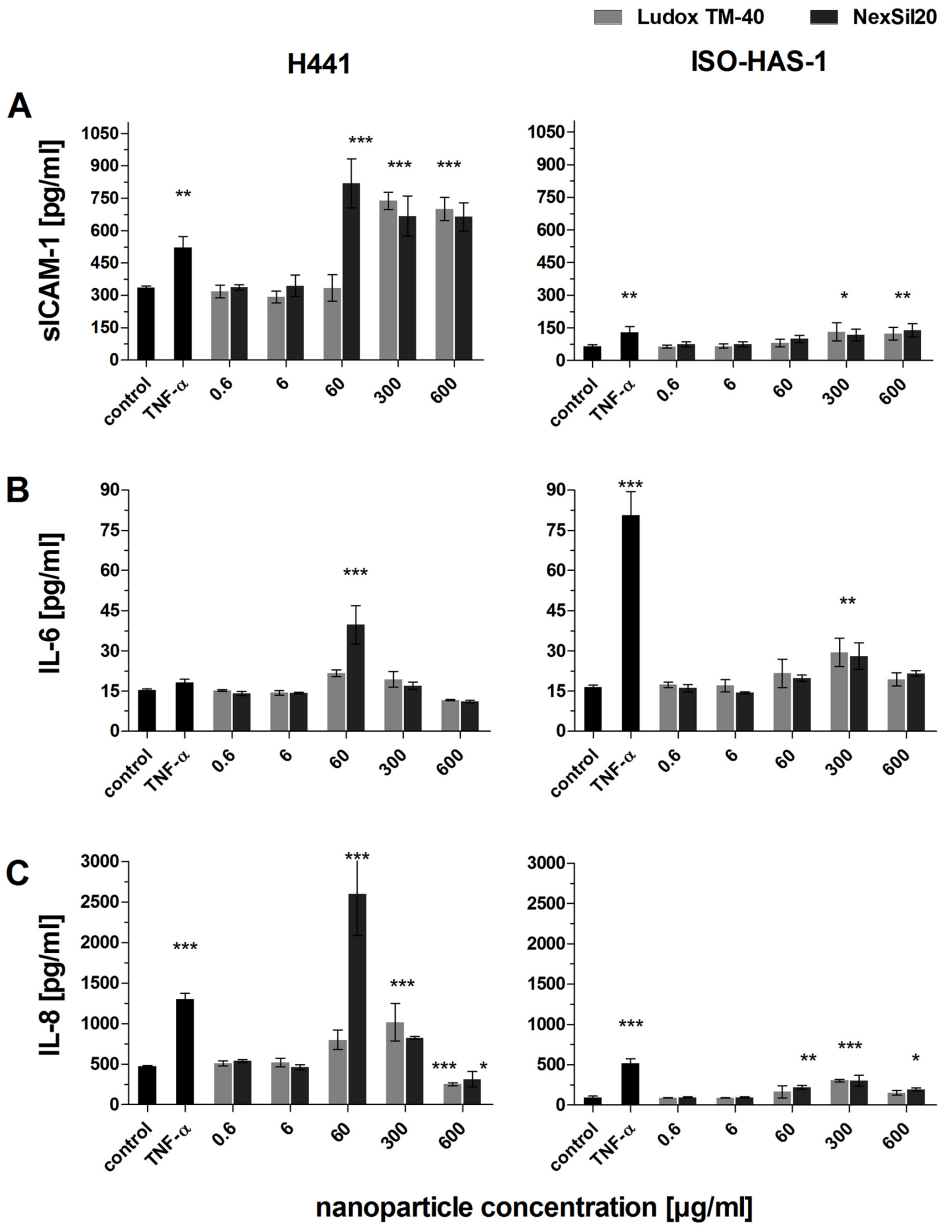
**The release of inflammatory mediators was measured after aSNP (Ludox TM-40: light grey, NexSil20: dark grey) exposure of monocultures of H441 and ISO-HAS-1 on 96 well plates (conventional monoculture)**. After 4 h incubation, aSNPs were removed and the cells were cultivated for further 20 h to detect sICAM-1, IL-6 and IL-8 release. Data are depicted as means ± S.D. of one representative experiment out of three independent experiments with n = 3 samples for each treatment. All independent experiments showed a comparable reaction following aSNP treatment. *P < 0.05, ** P < 0.01 and *** P < 0.001 compared to the untreated control

The release of inflammatory mediators measured after aSNP exposure of conventional monocultures on 96 well plates is shown in figure 3 for H441 and ISO-HAS-1 cells. For H441 cells even a concentration of 60 μg/ml NexSil20 induced a maximum release of sICAM-1 (Figure 3A). Concentrations of 300-600 μg/ml of both aSNPs show a 2fold increase of sICAM-1 for both cell lines compared to untreated controls (Figure 3A). Both aSNPs induced a maximum IL-6 release at concentrations of 60 μg/ml for H441 (Ludox TM-40: 1.4fold, NexSil20: 2fold) and 300 μg/ml for ISO-HAS-1 (2fold increase compared to untreated control) (Figure 3B). For both cell lines IL-8 release was seen at similar aSNP concentrations as for IL-6 (Figure 3C).

**Figure 4:**
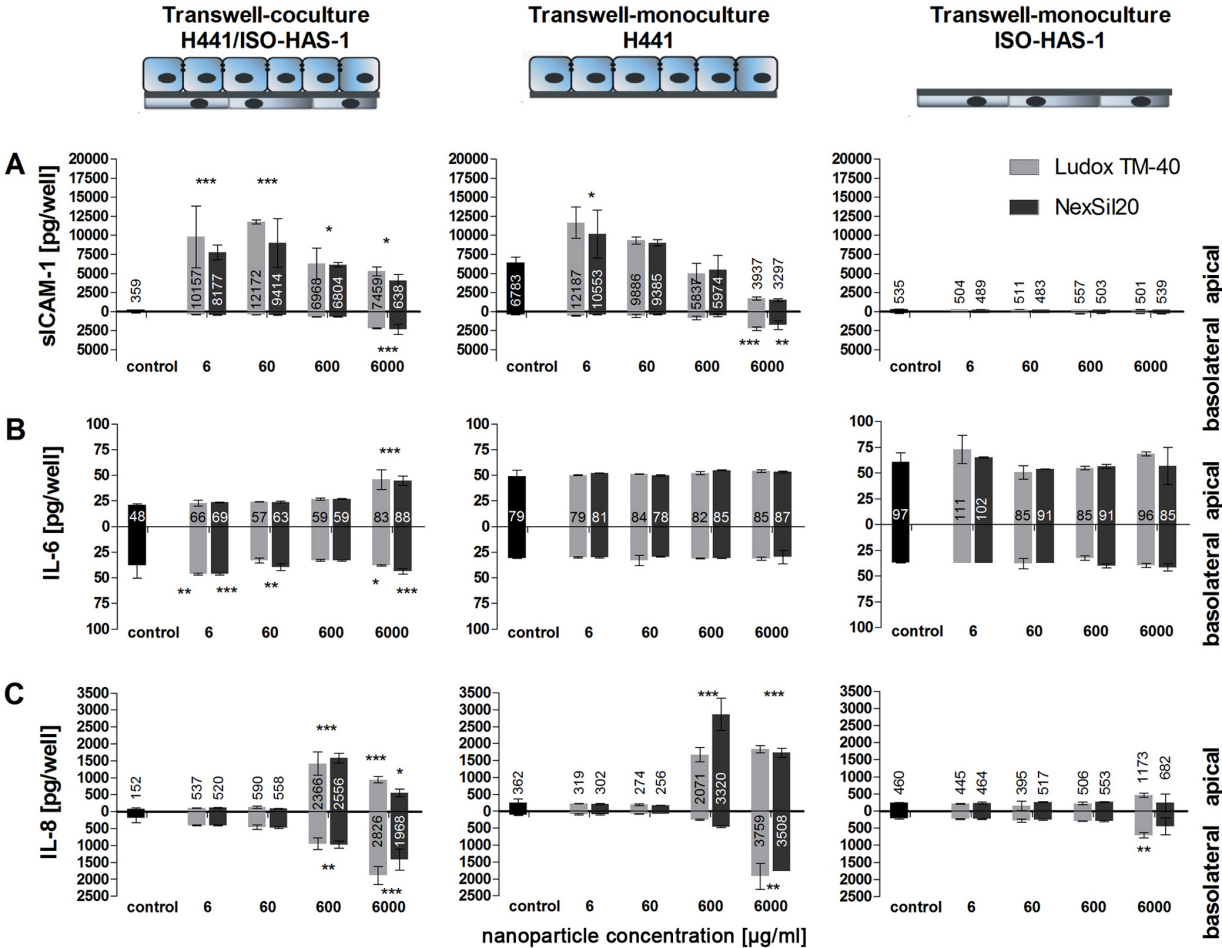
**The release of inflammatory mediators (sICAM-1, IL-6 and IL-8) is shown after aSNP (Ludox TM-40: light grey, NexSil20: dark grey) incubation on apical/basolateral differentiated cocultures of H441 and ISO-HAS-1, as well as on their appropriate monocultures grown on HTS 24-Transwell**^® ^**filters**. Cells were exposed to aSNPs from the apical side of the filter membrane to mimic an inhalative exposure to aSNPs. After 4 h serum-free incubation, aSNPs were removed and the cells were cultivated for further 20 h. Subsequently, medium supernatant of both compartments (apical: upper well, basolateral: lower well) was examined. Data are depicted as means ± S.D. of one representative (of three independent) experiments with n = 3 samples for each treatment. All independent experiments showed a comparable reaction following aSNP treatment. Exclusively in coculture, an apical exposure of 6 and 60 μg/ml aSNP caused an increased IL-6 and IL-8 release into the lower well (basolateral/endothelial side). For both monocultures increased amounts of sICAM-1, IL-6 and IL-8 in the lower well were not detected below concentrations of 600 μg/ml aSNP. *P < 0.05, ** P < 0.01 and *** P < 0.001 compared to the untreated control

Due to the different compartments that are formed by growing barrier-forming cells on transwells not only an apical/basolateral differentiated coculture but also polarized monocultures were grown on transwells (see Figure 4). These transwell-monocultures of H441 and ISO-HAS-1 with the same culture conditions and seeding surface serve as a control for the effects of an upper well aSNP exposure on the individual cell types. The analysis of sICAM-1 of the untreated controls of the three approaches (H441/ISO-HAS-1 (upper/lower surface), H441 (upper surface) and ISO-HAS-1 (lower surface) yielded significantly different basic levels of sICAM-1. The transwell-monoculture of H441 released in total 19fold more sICAM-1 compared to the coculture in total. The sICAM-1 response of the cocultures and transwell-monocultures of H441 to aSNPs with a value of 6 μg/ml was most intense (30fold in total) and decreased with increasing aSNP concentration. At concentrations from 6-600 μg/ml aSNPs there was no significant release of sICAM-1 into the lower compartment. With 6000 μg/ml aSNP sICAM-1 increased significantly in the lower well for the cocultures and the transwell-monocultures of H441. sICAM-1 response of the cocultures behaved in similar fashion to the H441 monoculture. Monocultures of ISO-HAS-1 growing at the lower surface of the transwell filter showed no sICAM release upon upper well exposure.

In coculture the IL-6 level rose slightly with increasing aSNP concentration whereas the transwell-monocultures did not show a clear response. In the lower well (basoloateral compartment) increased IL- 6 and IL-8 amounts were already detectable at low concentrations of aSNPs (6 μg/ml). The H441 in transwell-monoculture showed no basolateral release of either IL-6 or IL-8 at this concentration. In addition, the ISO-HAS-1 in transwell-monoculture were not affected by aSNPs concentrations of 6 μg/ml. At concentrations of 600 μg/ml aSNPs an IL-8 release (in total approx. 1.6fold of untreated control) into both compartments was detected for H441 in transwell-monoculture. The transwell-monoculture of ISO-HAS-1 showed no significant reaction to aSNP exposure from the upper well (basolateral exposure for the endothelial cells on the lower surface of the transwell) regarding IL-6 release. Not until concentrations of 6000 μg/ml aSNPs was there a slightly increased IL-8 release in the upper and lower well of ISO-HAS-1 in transwell-monoculture.

#### Transbilayer Electrical Resistance Measurement

**Figure 5:**
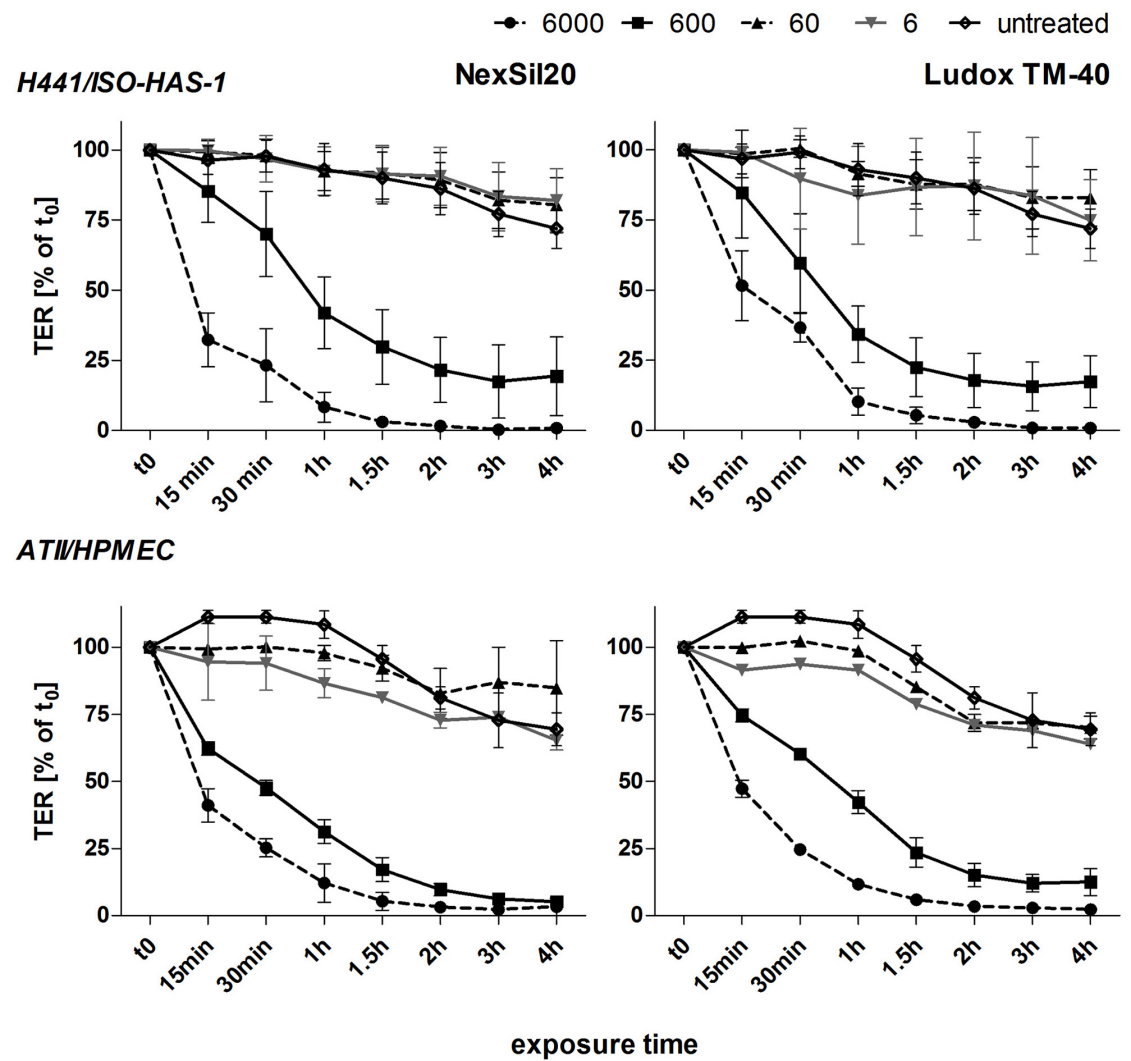
**Transmembrane electrical resistance was measured for cocultures of H441 with ISO-HAS-1 (H441/ISO-HAS-1) as well as for cocultures of primary isolated cells (alveolar type II and HPMEC (ATII/HPMEC))**. During 4 h incubation with aSNPs (NexSil20, Ludox TM-40 at concentrations of 6, 60, 600, and 6000, μg/ml) TER-values are depicted as % of time-point t0 (TER-value prior to aSNP treatment). Results are shown as means ± S.D. of 3 independent experiments with n = 2 samples for each treatment. For statistical analysis using Dunnett's Multiple Analyzing test, the 4 h value of the untreated samples was used as control. Treatment with 600 and 6000 μg/ml of both aSNP revealed a time-dependent decrease of TER after 4 h incubation. *P < 0.05, ** P < 0.01 and *** P < 0.001 compared to the untreated control

As shown in figure 5 the TER of untreated controls cultivated in parallel with the aSNP-stimulated groups over 4 hours decreased from t_0 _to t_4h _to approximately 70% (72 ± 7%) due to the change from serum-containing to serum-free medium. Due to the reduced osmolarity of the serum-free medium compared to the medium containing serum a slight decrease of the TER value can be expected. For both aSNPs (NexSil20 and Ludox TM-40) treatment with 6-60 μg/ml aSNPs gave similar results to the untreated control, while 600 μg/ml aSNP caused a reduction of TER to approximately 20% (Ludox TM-40: 17.4 ± 9% and NexSil20: 17.9 ± 13%) of the initial TER-value at t_0_. A concentration of 6000 μg/ml caused a complete disruption of the barrier after 2 h exposure for both aSNPs. Additionally, the effect of aSNPs on TER of primary isolated pulmonary microvascular endothelial cells in coculture with human alveolar type II/type I-like cells was studied as gold standard model of the alveolocapillary barrier. The behaviour of both cocultures, that is the cell lines (H441/ISO-HAS-1) and also the primary cells (ATII/HPMEC), upon aSNP exposure was comparable (600 μg/ml reduced TER to 12.5 ± 5% for Ludox TM-40 and to 6 ± 0.9% for NexSil20).

#### Morphological description of the cocultures before and after NP exposure

**Figure 6:**
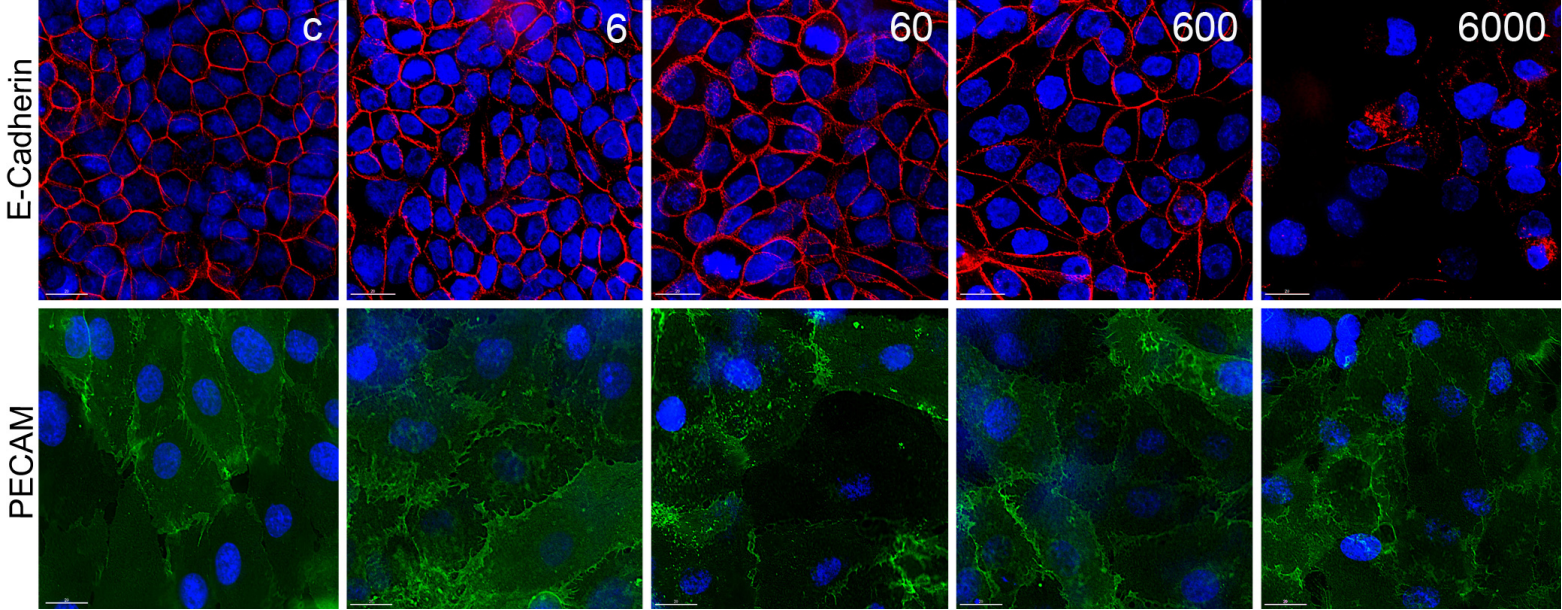
**After aSNP exposure layer integrity of H441 and ISO-HAS-1 in coculture was determined by immunofluorescent localization of junction-associated proteins**. The H441 cells of the coculture were incubated with aSNP (NexSil20: concentration range 0.6 - 6000 μg/ml, c: untreated control) for 4 h in serum-free medium. aSNPs were then removed and cells were cultivated for further 20 h. The H441 were labelled for E-cadherin, the ISO-HAS-1 were counterstained for PECAM-1.

In conventional monoculture morphological alterations were observed at an aSNP concentration of 600 μg/ml for both cell lines H441 and ISO-HAS-1 (see additional file 2 and 3: Figure S2 and S3). 60 μg/ml did not show any changes as a result of the aSNP treatment. Surprisingly, up to a concentration of 600 μg/ml aSNP the E-Cadherin staining pattern of the H441 in coculture showed no difference compared to the untreated control (Figure 6), even though the TER value decreased significantly at this dose (Figure 5). A concentration of 6000 μg/ml aSNPs, a factor 10 higher than in monoculture, caused visible changes in the assembly of cell-cell junctions and first signs of cell detachment (Figure 6). These findings explain the complete breakdown of TER resulting in apically released cytokines passing through the barrier and being basolaterally detectable. The indirectly exposed ISO-HAS-1 (PECAM-1 counterstaining) did not show any morphological alterations even at a concentration of 6000 μg/ml.

#### Effect of aSNP on apoptosis markers

**Figure 7:**
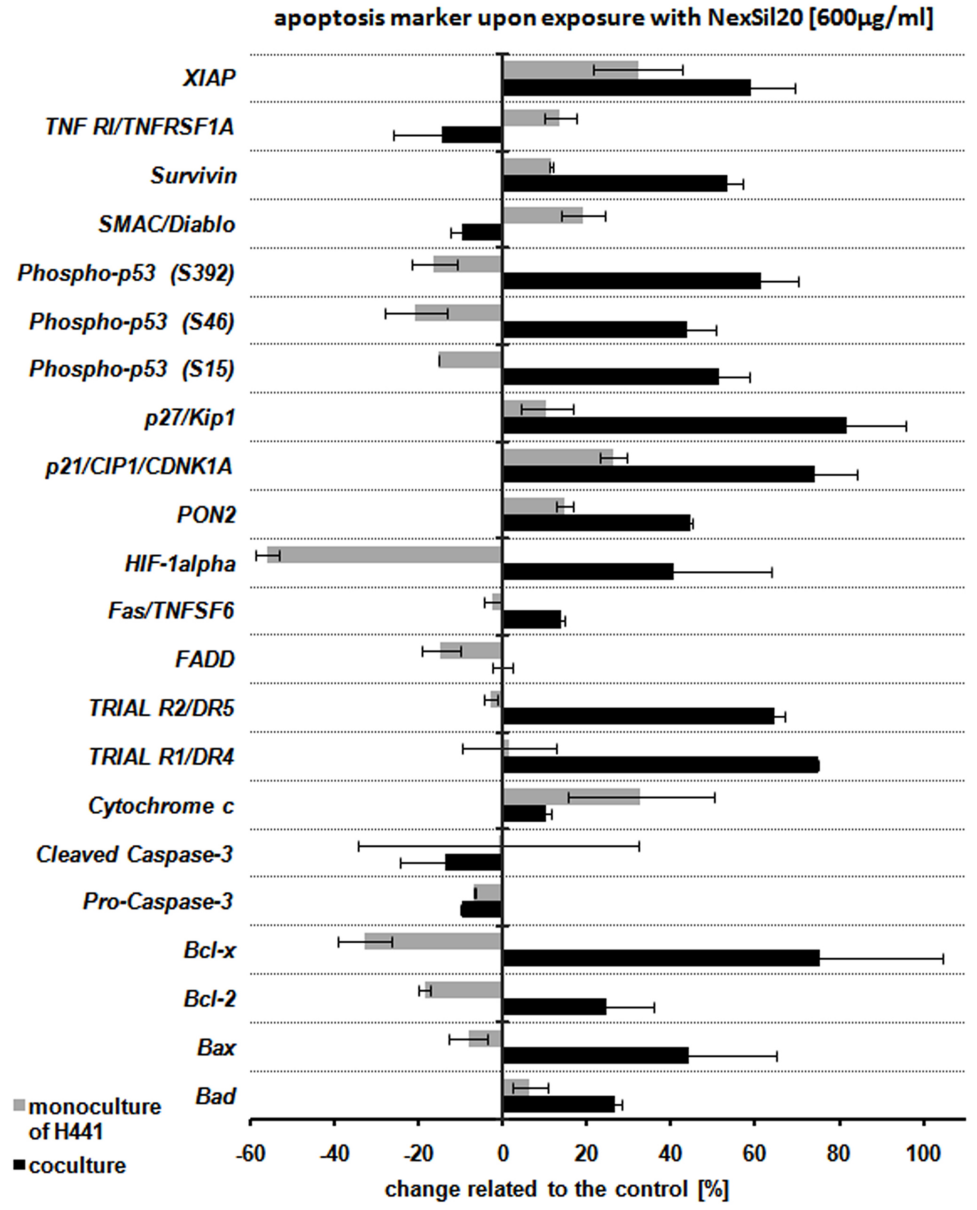
**Protein expression of cell death regulators was analysed after apical exposure of differentiated cocultures with aSNPs (NexSil20: 600 μg/ml) to mimic the situation after accidental inhalation of aSNPs**. After 4 h incubation with aSNPs filter membranes were excised, lysed for 30 min and an apoptosis protein array was performed. Data are depicted as means ± S.D. from 2 independent experiments with n = 4 samples for each treatment. Quantitative analysis of the array revealed increased levels of a number of apoptosis markers, especially of the intrinsic pathway.

The protein array for apoptosis markers demonstrated for the coculture that the expression of a number of intrinsic apoptosis markers was elevated in a time-dependent way after exposure to 600 μg/ml NexSil20 (data for 10 min and 1 h exposure are not shown). Figure 7 shows the percentage changes of apoptosis marker protein content related to the non-treated control after 4 h exposure. The assay revealed a phosphorylation of the p53-protein at Ser15, Ser46 and Ser392 (Ser15: 151 ± 7%, Ser46: 143 ± 7%, Ser392: 161 ± 9%). An increase of pro-apoptotic markers such as Bad (126 ± 1.4%) and Bax (144 ± 20.5%) and its ligands was also detected. In addition, the assay detected cytochrome c release (110 ± 0.9%) into the cytosol. Further major apoptotic markers, such as Fas (113 ± 0.6%), DR4 (174 ± 0.4%) and DR5 (164 ± 2.4%) were also upregulated. Concerning hypoxia, HIF-1α (140 ± 23%), an hypoxia inducible factor, showed a 1.4 fold increased level compared to the untreated control. Additionally, an on average 1.8 fold higher amount of cell cycle and proliferation regulator proteins p21 (174 ± 10%) as well as p27 (181 ± 13.8%) was observed compared to the untreated control. However, elevated levels of apoptosis inhibitory factors such as XIAP (X-linked Inhibitor of apoptosis Protein: 158 ± 10.8%) and survivin (153 ± 3.6%) were indicated by this array following NexSil20 exposure. The increase of paraoxonase-2 (Pon2: 145 ± 0.4%), which has a reductive function to reactive oxygen species, is an indication that the cells of the coculture are in a stressed condition after 4 h apical exposure to 600 μg/ml NexSil20.

## Discussion

Monocultures on tissue culture plastic are mostly used to study cytotoxicity and uptake of nanoparticles (NPs) in target cells. To investigate functional effects on biological diffusion barriers we investigated the effects of amorphous silica nanoparticles (aSNP) in a polarized barrier-forming coculture model compared to monocultures grown under conventional culture conditions. To detect aSNP cytotoxicity viability (MTS) and membrane integrity (LDH release) tests were performed. A good correlation of the MTS assay and LDH assay following aSNP treatment was observed for conventional monocultures. By contrast, the cocultures were less sensitive on aSNP exposure with respect to LDH release. Comparable to the results found for morphological changes, the responsiveness towards aSNP treatment was shifted from an aSNP concentration of 600 μg/ml in monoculture to 6000 μg/ml in coculture (factor 10). Under conventional tissue culture conditions monocultures of H441 show a fragmented immunostaining of tight junctional (TJ) and adherens junction (AJ) proteins, whereas polarized cells (apical/basolateral differentiation) in coculture establish a functional TJ and AJ network (see additional file 4: Figure S4) [9]. The higher responsiveness towards amorphous silica toxicity by the non-polarized conventional monoculture may be due to the higher surface area accessible to the aSNPs. For the cocultures another sensitive marker for toxic effects is the measurement of transbilayer electrical resistance (TER). For both aSNPs a concentration of 6000 μg/ml resulted in a complete breakdown of the TER for H441/ISO-HAS-1. A similar behaviour was seen in preliminary experiments with cocultures of primary alveolar epithelial (ATII) and endothelial cells (HPMEC), which is the gold standard model of the lower respiratory tract. While LDH measurement at a concentration of 600 μg/ml aSNPs resulted only in an insignificant LDH release, the TER-values decreased significantly. These data indicate that the TER measurement is a highly sensitive method to evaluate slight changes in barrier properties before any toxic effects are detectable.

Despite their toxicity aSNP induced a significant pro-inflammatory response (sICAM-1, IL-6, IL-8) in epithelial and endothelial cells in conventional monocultures as well as in the polarized monoculture on transwell filter units. ICAM-1 (intercellular adhesion molecule-1) is a crucial mediator for the attraction and adhesion process of endothelial as well as epithelial cells to leukocytes. It is involved in numerous lung diseases, such as asthma [16], ARDS (acute respiratory distress syndrome) [17] and ALI (acute lung injury) [18]. Direct stimulation of rat lung tissue by pathogens causes an upregulation of ICAM-1 in the bronchiolar epithelial cells, ATII, and in vascular endothelial cells [19]. Furthermore, increased soluble ICAM-1 levels were found in the bronchoalveolar fluid of mice and increased ICAM-1 expression was detected on alveolar macrophages and ATII cells exposed to ultrafine crystalline silica particles [20,21]. For H441 cells in conventional monoculture a concentration of 60 μg/ml NexSil20, which showed no effects in MTS and LDH assays, induced a 3fold increased sICAM-1 release compared to the untreated control. Nevertheless, the responses of the monocultures differed markedly from that observed in cocultures. In coculture an inflammatory response was already detectable at much lower aSNP concentrations than in conventional monoculture. Upper well exposure to 6 μg/ml resulted in a 10fold higher sICAM-1 release to the upper well compared to monocultures. Additionally, in coculture low concentrations of 6-60 μg/ml aSNP, which had no effect on TER and LDH, resulted in an IL-6 and IL-8 release to the lower compartment, whereas no increase of both cytokines was measured on the exposed side. By contrast, the polarized transwell-monocultures of H441 showed no changed basolateral release of both cytokines. Additionally, experiments with a fluorescent aSNP (SicaStar Red 30 nm, micromod, unpuplished) revealed that particles, when apically applied (H441), do not reach the Iso-Has-1 seeded on the bottom side. By an apically exposure without H441 on the top side however the aSNP crossed the filter membrane and were detected in the Iso-Has-1. Thus, it seems that not aSNPs transported to the ISO-HAS-1 cells but crosstalk between the aSNP-stimulated H441 and the ISO-HAS-1 resulted in the increased IL-6 and IL-8 release to the lower compartment. An interesting question would be which proportion of cytokines being detectable basolaterally is due to the ISO-HAS-1 in co-culture, and how much is due to breakdown of the barrier? With this experimental design used so far it was not possible to answer this question. Answers should have been given by exposing monocultures on transwells to the aSNPs. In these monocultures no cytokine release was detected at low concentrations of aSNPs. Although there was no breakdown of TER a clear basolateral increase of cytokines was detected at low concentrations of aSNPs in the coculture. We assume that a crosstalk of both cell types in coculture is needed to evoke a basolateral cytokine secretion at low concentrations of aSNPs. The pro-inflammatory cytokines IL-6 and IL-8, which play a decisive role in the recruitment and regulation of neutrophils, have been shown to be involved in the NP-induced inflammatory reaction by other groups [22,23].

A functioning cytokine network across the alveolar-capillary barrier is pivotal for cellular communication during pulmonary inflammation. The polarized coculture allows a compartmentalisation with two biological barrier components apically facing the different compartments. This assembly further enables communication of both the epithelial and endothelial cells, although initially only the exposed epithelial cells might have contact with the nanoparticles but the indirectly affected endothelial cells forward the signal of inflammation. Since the lung is composed of many different cell types, it is of great concern to conduct cytotoxicological studies with complex coculture systems consisting of more cell types, consequently allowing cellular communication rather than using monocultures with only one single cell type. Several studies focusing on nanoparticle exposed human epithelial airway coculture models have already been performed to address this issue [24-27]. Müller et al [26] revealed an altered response of IL-8 release following particle exposure on a triple cell coculture of A549 with MDMs (monocyte-derived macrophages) and MDDCs (Monocyte-derived dendritic cells) compared to the respective monocultures. Wottrich et al. [27] showed an increased sensitivity to particles with respect to cytokine release (IL-6, IL-8) from human epithelial cells A549 in coculture with macrophages, compared to the cells in monoculture. Corroborating these findings our study additionally addressed the barrier properties of the alveolar-capillary barrier and demonstrated changes in a model in which a functional barrier is established.

The results of the apoptosis array show that the finely tuned balance of pro-apoptotic and anti-apoptotic factors which usually exists in healthy tissue is disturbed after exposure to 600 μg/ml aSNPs. A number of apoptosis markers are elevated at 4 h aSNP exposure in coculture. The detected increase of HIF-1α, hypoxia inducible factor and the phosphorylation of the p53-protein at Ser15, Ser46 and Ser392 is a sign of DNA-damage or hypoxia due to the amorphous silica exposure. In keeping with the p53 activation increases of pro-apoptotic markers, contributing to the intrinsic pathway, such as Bad and Bax, with its counterparts, Bcl-2 and Bcl-x, and a concomitant increase in cytochrome c release, as well as the TRIAL receptor KILLER/DR5 were detected. Interestingly, XIAP (X-linked Inhibitor of apoptosis Protein) a member of the Inhibitor of apoptosis family was also upregulated following aSNP exposure. Since this protein binds and inhibits caspase 3, 7 and 9 this could explain why neither the procaspase nor the cleaved active product of caspase 3 was increased.

Numerous cytotoxicity studies also indicate that DNA-damage or hypoxia and the subsequent mitochondria-regulated apoptosis are triggered by silica nanomaterials [28-32]. In an *in vivo *study on rats, high administered doses of amorphous silica to the lung led to an increased cytotoxicity (LDH release in lung lavage), necrosis or apoptosis (increased TUNEL staining of epithelial cells) [33]. An induction of ER-stress, which itself can initiate apoptotic processes [34-36], can be assumed by the upregulation of the paraoxonase 2 (PON2) after 600 μg/ml aSNP treatment. For the H441 alone (monoculture on transwells) some apoptotic markers such as i.e. p53, HIF-1α, or Bcl-x, Bcl-2 and Bax do not seem to be affected due to aSNP treatment. This might be an indication that the apoptotic response might originate from the indirectly exposed ISO-HAS-1 situated on the lower aspect of the filter membrane. This might be a further indication of cross-talk between the directly aSNP-exposed H441 and the ISO-HAS-1 at the other side of the filter membrane.

## Conclusion and prospects

As our results show, the behaviour of the conventional monocultures with respect to cytotoxicity, such as membrane integrity (LDH), inflammation (cytokines) and barrier property measurements (Transbilayer Electrical Resistance) is markedly different from that of polarized cells in a relevant coculture system and does not take into account an interplay of adjacent cells, so-called cellular crosstalk.

Further efforts need to be made to evaluate if the cytotoxic effects of the aSNPs are related to the internalized amount of the aSNPs. The quantification of internalized aSNP may be correlated to the observed dose-dependent cytotoxic effects in mono- and coculture. First studies using fluorescently labelled aSNPs show that the aSNPs enter both the epithelial and the endothelial cell types, but each to a different extent. Additionally, a first correlation of the internalized amount of aSNP and the observed cytotoxic effects could be made.

## Abbreviations

AJ: adherens junction; aSNP: amorphous silica nanoparticle; ATII: alveolar type II cells; HPMEC: human pulmonary microvascular endothelial cells; H441: lung adenocarcinoma cell line NCI H441; IL-6: Interleukin 6; IL-8: Interleukin 8; ISO-HAS-1: clone of the angiosarcoma cell line ISO-HAS; LDH: lactate dehydrogenase; TER: trans-monolayer or -bilayer electrical resistance; TER t_0_: TER value before aSNP exposure; TJ: tight junction; sICAM-1: soluble intercellular adhesion molecule-1

## Competing interests

The authors declare that they have no competing interests.

## Authors' contributions

The experiments described herein were carried out by JK in partial fulfilment of the requirements for a biological doctoral degree at the Johannes Gutenberg University, Mainz, Germany. JK and MIH have made substantial contributions to conception and design as well as analysis and interpretation of the data. Furthermore JK and MIH have drafted the manuscript. CB carried out the experiments concerning characterisation of the nanoparticle solutions and the following interpretations and draftings. Herein MM has given substantial input concerning conception of the analysis, following interpretations and critically revision for intellectual content. CP has given intellectual input concerning cell culture and the apoptosis analysis and participated in the revision of the manuscript. As coordinator of the cluster BIONEERS within the DFG priority program SPP 1313 RS contributed in discussions concerning the experimental design and has given substantial intellectual support to the studies. REU has given substantial intellectual support concerning the conception and revision of the manuscript and has given final approval of the version to be published. CJK has contributed substantially to the development of the coculture model and revised the manuscript critically for important intellectual content. All authors read and approved the final manuscript.

## Supplementary Material

Additional file 1 Figure S1: The fact that the size of the nanoparticles in the dry state (*D*_*TEM*_) was nearly the same as in solution indicates that the effect of particle shrinking during the preparation of the samples for TEM is minimal. Click here for file

Additional file 2 **Figure S2: After aSNP exposure, H441 in conventional monoculture were checked for morphological alterations.** The cells were incubated with aSNP (NexSil20: concentration range 0.6 - 6000 μg/ml, c: untreated control) for 4 h in serum-free medium. aSNPs were then removed and cells were cultivated for further 20 h. Additionally, cells were counterstained for F-actin with Phalloidin-TRITC. Visual examination was conducted by means of a fluorescent microscope (personalDV, Applied Precision, Issaquah, USA). Click here for file

Additional file 3 **Figure S3: After aSNP exposure, ISO-HAS-1 in conventional monoculture were studied for morphological alterations.** The cells were incubated with aSNP (NexSil20: concentration range 0.6 - 6000 μg/ml, c: untreated control) for 4 h in serum-free medium. aSNPs were then removed and cells were cultivated for further 20 h. Additionally, cells were counterstained for F-actin with Phalloidin-TRITC. Visual examination was conducted by means of a fluorescent microscope with DIC (personalDV, Applied Precision, Issaquah, USA). Click here for file

Additional file 4 **Figure S4: Comparison of the H441 in conventional monoculture and in coculture (with ISO-HAS-1) regarding the development of tight junctional TJ (ZO-1) and adherens junctional structures (β-Catenin and E-Cadherin).** Under conventional tissue culture conditions monocultures of H441 show a fragmented immunostaining of tight junctional (TJ) and adherens junction (AJ) proteins, whereas polarized cells in coculture establish a functional TJ and AJ network. Visual examination was conducted by means a fluorescent microscope (personalDV, Applied Precision, Issaquah, USA). Click here for file
