# Supplementary figures and images for: Inflammatory and cytotoxic responses of an alveolar-capillary coculture model to silica nanoparticles: Comparison with conventional monocultures
Source: Part Fibre Toxicol. 2011 Jan 27;8:6. doi: 10.1186/1743-8977-8-6 (PMC3040689; doi:10.1186/1743-8977-8-6)

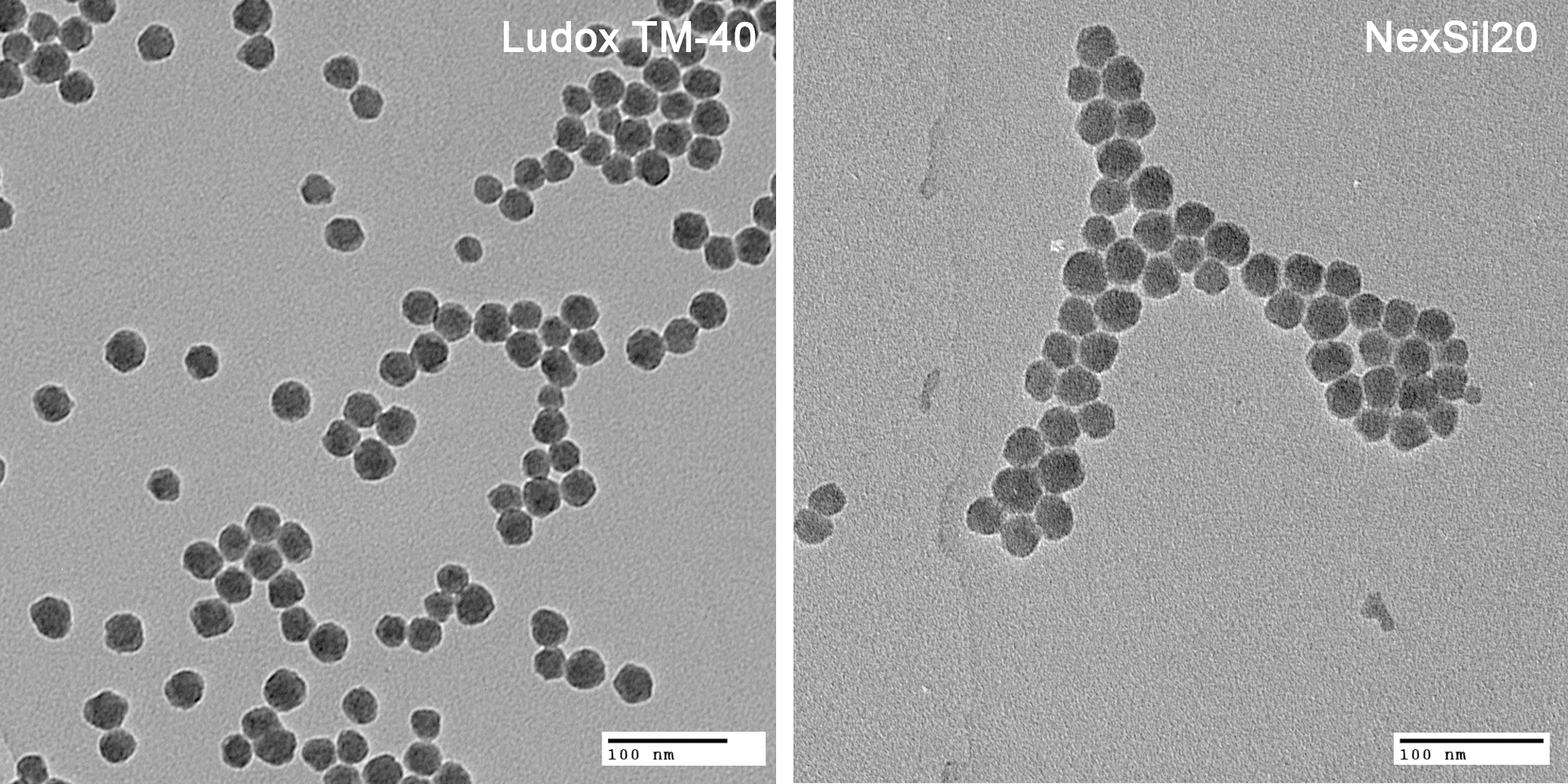

Supplement: Additional file 1 — Figure S1: The fact that the size of the nanoparticles in the dry state (DTEM) was nearly the same as in solution indicates that the effect of particle shrinking during the preparation of the samples for TEM is minimal. [file 1743-8977-8-6-S1.JPEG]

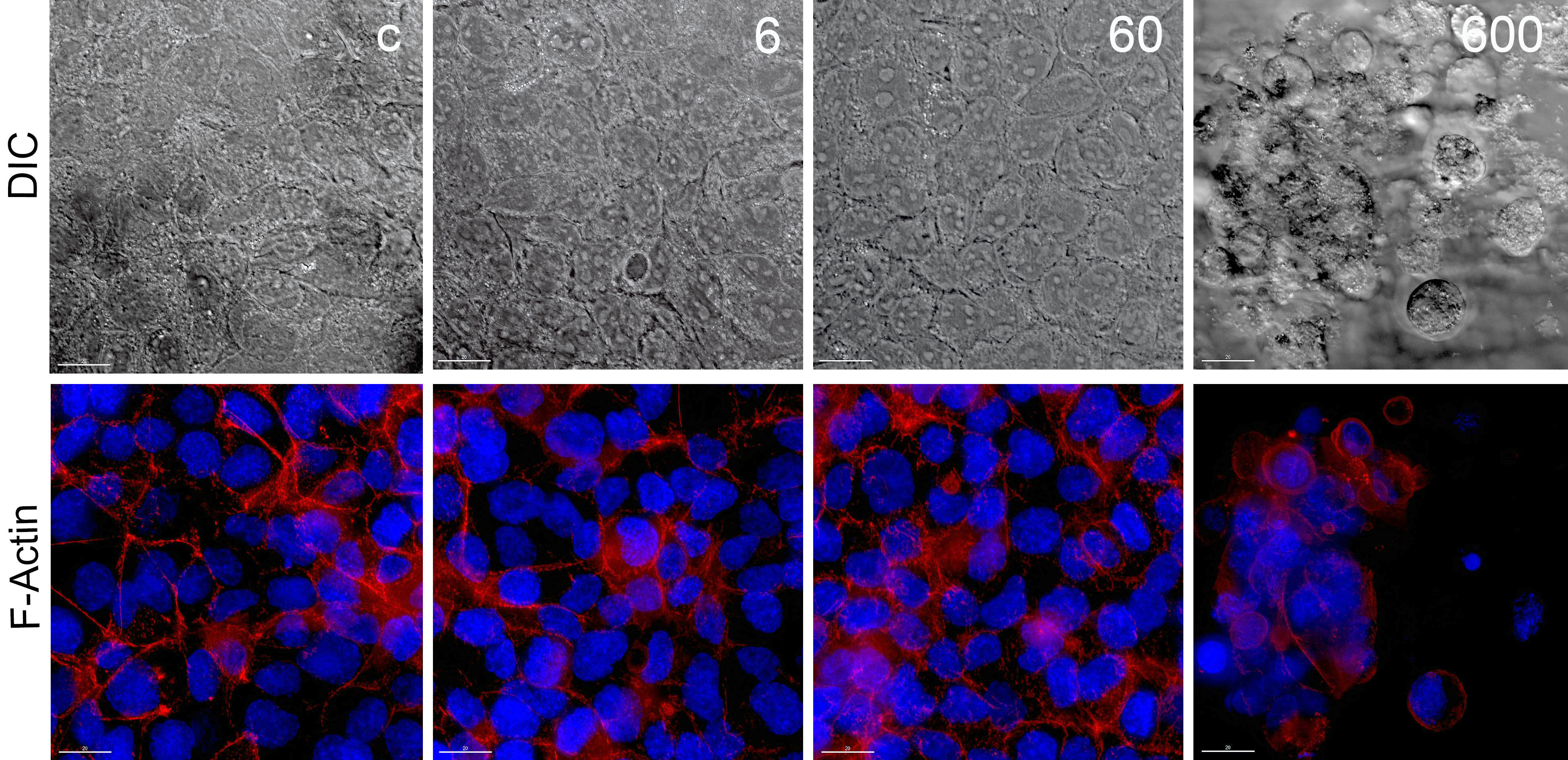

Supplement: Additional file 2 — Figure S2: After aSNP exposure, H441 in conventional monoculture were checked for morphological alterations. The cells were incubated with aSNP (NexSil20: concentration range 0.6 - 6000 μg/ml, c: untreated control) for 4 h in serum-free medium. aSNPs were then removed and cells were cultivated for further 20 h. Additionally, cells were counterstained for F-actin with Phalloidin-TRITC. Visual examination was conducted by means of a fluorescent microscope (personalDV, Applied Precision, Issaquah, USA). [file 1743-8977-8-6-S2.JPEG]

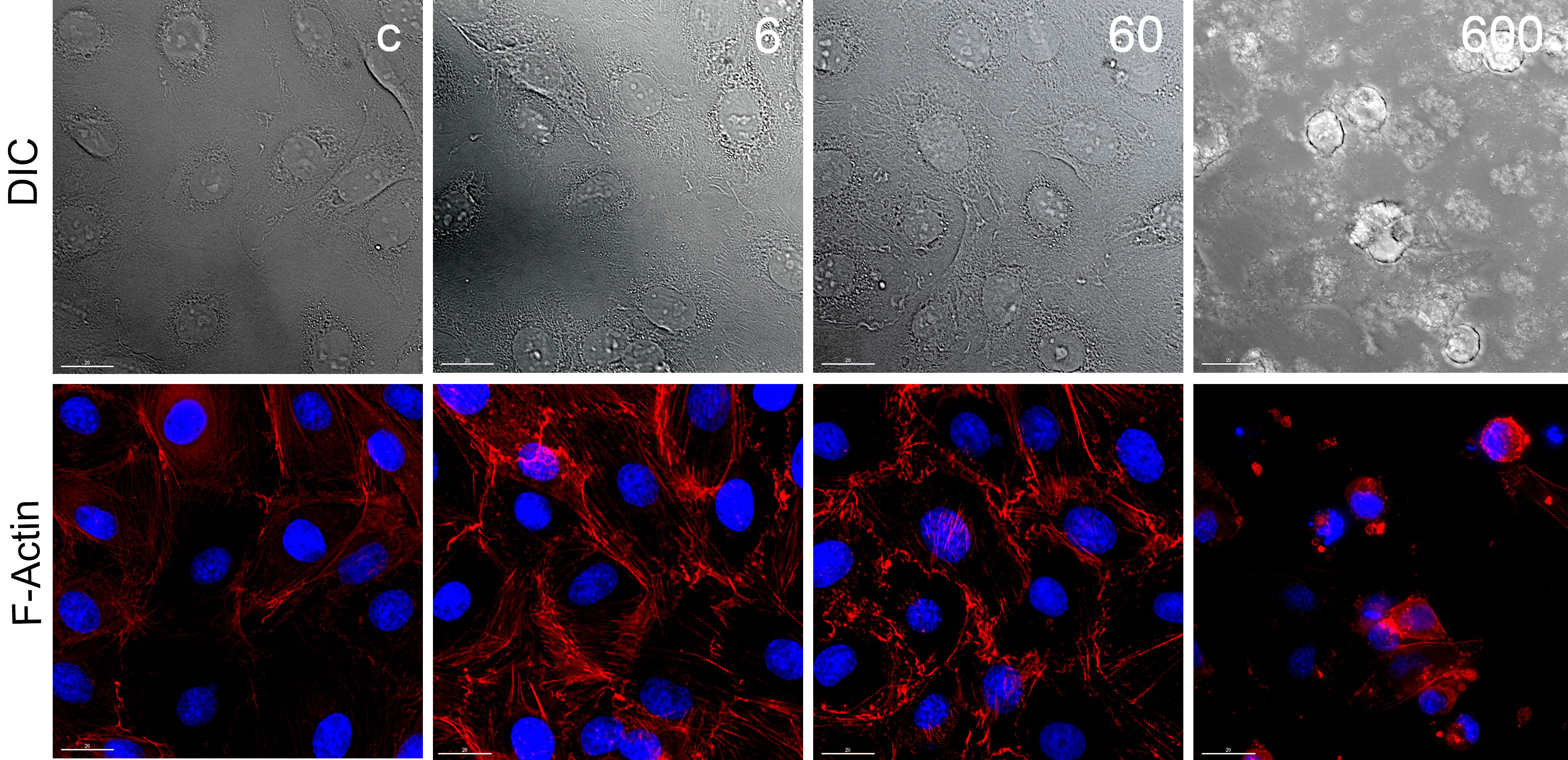

Supplement: Additional file 3 — Figure S3: After aSNP exposure, ISO-HAS-1 in conventional monoculture were studied for morphological alterations. The cells were incubated with aSNP (NexSil20: concentration range 0.6 - 6000 μg/ml, c: untreated control) for 4 h in serum-free medium. aSNPs were then removed and cells were cultivated for further 20 h. Additionally, cells were counterstained for F-actin with Phalloidin-TRITC. Visual examination was conducted by means of a fluorescent microscope with DIC (personalDV, Applied Precision, Issaquah, USA). [file 1743-8977-8-6-S3.JPEG]

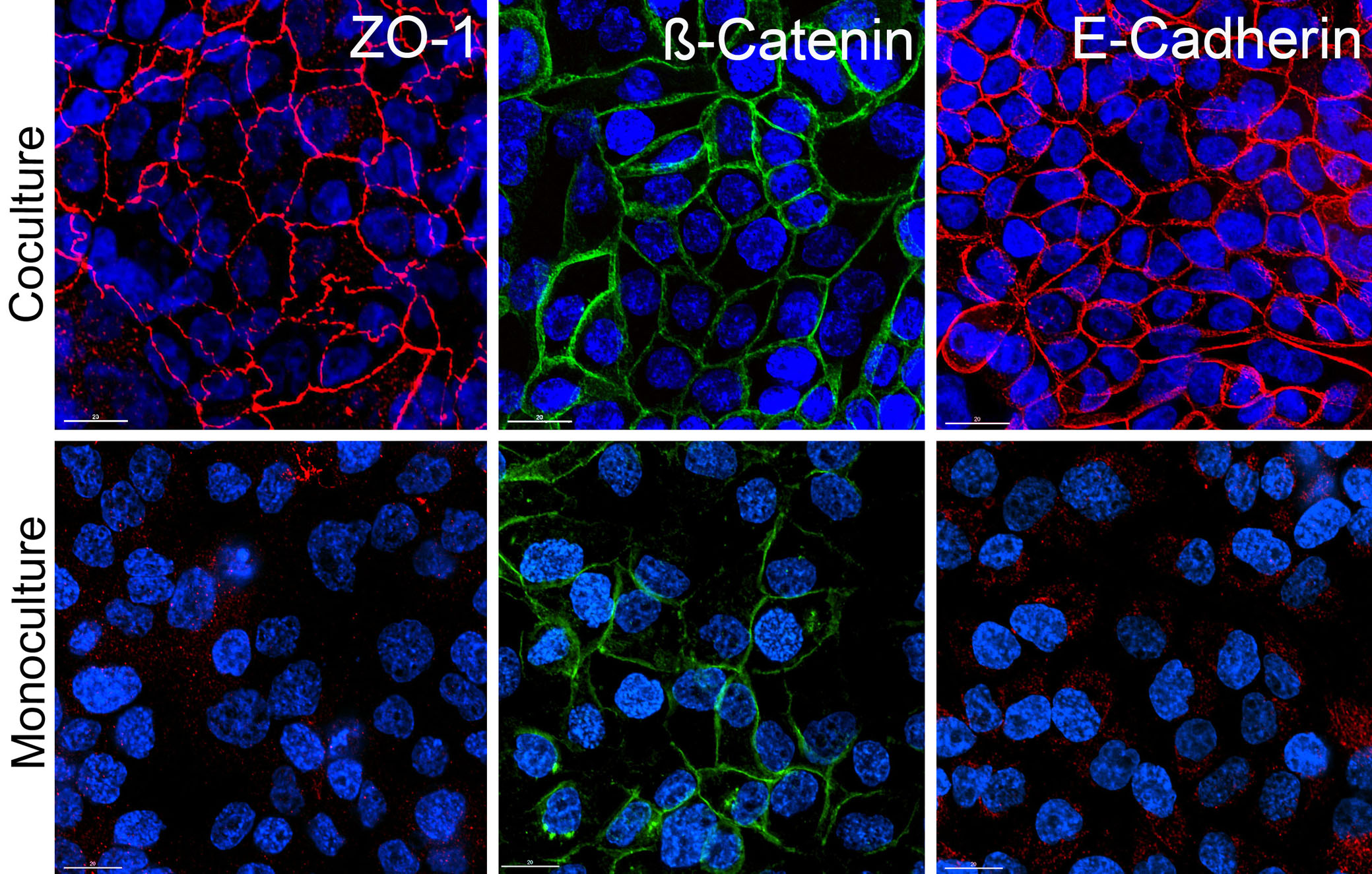

Supplement: Additional file 4 — Figure S4: Comparison of the H441 in conventional monoculture and in coculture (with ISO-HAS-1) regarding the development of tight junctional TJ (ZO-1) and adherens junctional structures (β-Catenin and E-Cadherin). Under conventional tissue culture conditions monocultures of H441 show a fragmented immunostaining of tight junctional (TJ) and adherens junction (AJ) proteins, whereas polarized cells in coculture establish a functional TJ and AJ network. Visual examination was conducted by means a fluorescent microscope (personalDV, Applied Precision, Issaquah, USA). [file 1743-8977-8-6-S4.JPEG]
